# Dual Users and Electronic Cigarette Only Users: Consumption and Characteristics

**Published:** 2018-06

**Authors:** Alexander S. Lee, Joy L. Hart, Kandi L. Walker, Rachel J. Keith, S. Lee Ridner

**Affiliations:** Department of Communication, University of Louisville, Louisville, Kentucky, USA and American Heart Association Tobacco Regulation and Addiction Center, Dallas, Texas, USA; Department of Communication, University of Louisville, Louisville, Kentucky, USA and American Heart Association Tobacco Regulation and Addiction Center, Dallas, Texas, USA; Department of Communication, University of Louisville, Louisville, Kentucky, USA and American Heart Association Tobacco Regulation and Addiction Center, Dallas, Texas, USA; School of Medicine, University of Louisville, Louisville, Kentucky, USA and American Heart Association Tobacco Regulation and Addiction Center, Dallas, Texas, USA; College of Nursing, East Tennessee State University, Johnson City, Tennessee, USA

**Keywords:** Electronic cigarette, Dual use, Vape, Nicotine consumption, E-juice, Vape shop

## Abstract

**Background:**

E-cigarette use has grown in popularity, especially as the devices have been touted as smoking cessation tools. In an exploratory study, we sought to compare dual users (i.e., users of both combustible tobacco and e-cigarettes) to e-cigarette only users.

**Methods:**

The Electronic Cigarette Opinion Survey (ECOS) was employed to assess users' (n=78) perceptions and consumption of e-cigarettes and combustible cigarettes. Quantity of e-juice and nicotine used and time of initial nicotine exposure were assessed. Multivariable logistic regression was used to evaluate the association between e-cigarette use behaviors and being an e-cigarette only user compared to a dual user.

**Results:**

Compared to dual users, e-cigarette only users consumed higher levels of nicotine in e-juice (p=0.0009) and more nicotine per month (p=0.03). For dual users, the time of first nicotine exposure after waking was significantly earlier than for e-cigarette only users (mean= 9.6 minutes (SD= 8.0) and mean= 26.6 minutes (SD= 22.0), respectively; p=0.0056). Results from the regression models suggest the amount of e-juice consumed and time of first nicotine exposure after waking are significantly associated with being an e-cigarette only user.

**Conclusions:**

These findings shed light on the perceptions and use patterns of e-cigarette only users compared to dual users. As regulation of e-cigarettes is considered, understanding the impact of e-cigarettes and dual use is imperative. Despite frequent marketing claims that e-cigarettes are completely safe, health campaigns need to convey emerging and mixed findings on safety as well as current scientific uncertainty to the public.

## 1. Introduction

Overwhelming evidence on the negative health effects of smoking has led many combustible cigarette users to consider quitting. Toward this goal, some combustible cigarette users have switched to electronic cigarettes (e-cigarettes), an alternative nicotine delivery method, which many perceive as a more health conscious option. Some hope that e-cigarettes will be less addictive and help them quit or reduce their dependency on combustible cigarettes [1,2].

Given the relative newness of e-cigarettes, there is not yet a body of systematic research that chronicles the health effects, especially over the long-term, of inhaling vaporized substances, or “vaping”, and such research is needed [3]. Confusion and uncertainty about the health and safety of e-cigarettes abound, with some studies reporting positive health outcomes and others reporting negative ones. For example, e-cigarette users have reported less coughing and improved breathing [4]. Conversely, reported negative health effects include throat and mouth irritation and pulmonary and respiratory problems [5]. An assessment of 405 health effects reported by e-cigarette users revealed that 326 were negative. [5]

Adding to the controversy, many e-cigarette users and people considering e-cigarette use turn to social media to better understand the health effects of these products [6, 7]. Through Twitter, marketers tout the cessation help e-cigarettes offer [8] and, on YouTube, numerous videos depict e-cigarettes positively and as healthier than smoking combustible cigarettes [9]. In short, scientific investigation on the health effects of e-cigarettes continues, but amassing a body of evidence takes considerable time. In the interim, sharing information through social media is rapid and widespread, and often the information conveyed about e-cigarettes through these channels is highly positive, cultivating the perception that e-cigarettes are a health conscious alternative to combustible cigarettes [2, 10]. Smokers who have failed to quit may be drawn to the allure of fewer health risks and turn to e-cigarettes [2, 11]. Many e-cigarette users praise the devices for improving their health, with one study reporting that e-cigarette users called vaping “life-saving” [11].

For individuals interested in learning more about or trying e-cigarettes, vape store retailers are also a key source of information [1]. These retailers often promote e-cigarettes as a safer alternative to combustible cigarettes, listing numerous health benefits of e-cigarettes over combustible cigarettes [1, 12]. In general, the claims are that e-cigarettes do not have the same adverse health risks as combustible cigarettes and therefore are healthier to use [11]. However, given the lack of empirical evidence, many researchers question such claims, arguing that vape shop employees may be underestimating potential negative health effects and overestimating potential health benefits and/or cessation outcomes [13].

Although many combustible cigarette smokers seek out e-cigarettes for cessation purposes, many are unable to quit smoking entirely, instead becoming dual users or alternating between periods of combustible cigarette and e-cigarette use [14-18]. Despite cessation claims, e-cigarettes may instead perpetuate nicotine addiction, providing dual users more access to nicotine. With fewer restrictions on where they can be used, e-cigarettes may ease access to nicotine and thus allow increased nicotine consumption. To that end, some findings suggest an increased likelihood of dual use and decreased interest in quitting or limiting e-cigarette use when greater levels of nicotine are consumed in e-juice [19].

Despite scientific uncertainty on the health effects of e-cigarette use, positive views of e-cigarettes are common, often fueled through marketing efforts. Further, dual users and e-cigarette only users frequently share their viewpoints and experiences, often foregrounding positive experiences and health outcomes. To better understand the perceptions and behaviors of dual users and e-cigarette only users, we sought to compare e-cigarette use among two vape shop customer groups: dual users (currently using e-cigarettes and combustible cigarettes) and e-cigarette only users.

## 2. Methods

### 2.1. Data Collection

After approval was granted by the university’s Institutional Review Board, the Electronic Cigarette Opinion Survey (ECOS) questionnaire was distributed to customers in nine vape shops in Louisville, Kentucky. The vape shops were systematically selected across the city to capture a range in demographics. The ECOS questionnaire was investigator generated, contained 39 items, and examined socio-demographics, perceptions and opinions of e-cigarette users, as well as current and previous patterns for e-cigarette and other tobacco use. After securing permission from the vape shops to survey customers, members of the research team invited customers 18 years and older to complete ECOS. Questionnaires were answered in person in the shop, and it took participants approximately 10 minutes to complete responses. No incentives were given for participation. Items that were applicable to the dual use of tobacco and e-cigarettes or e-cigarette only use were used in the analysis.

### 2.2. Participants

Data from 80 participants were collected. Two participants were not included in the analysis because they were first time e-cigarette users. Most of the remaining 78 participants were male (n=58; females n=19; one person did not specify sex). The age range was 18.2 years to 58.8 years with a mean age of 31. Most participants were white (88.2%; n= 67) and employed, with a household income greater than $50,000 (n=43).

### 2.3. Measures and Definitions

#### 2.3.1. Socio-demographic Characteristics

Socio-demographic characteristics included gender, age, race, education and household income. For this analysis, education level was dichotomized into: not holding a college degree (GED, graduated high school, completed vocational training or some college education) and holding a college degree (2-year college degree, 4-year college degree or advanced/professional degree). Household income was median split at above or below $50,000 per year, which also reflects the median household income of the city.

#### 2.3.2. Dual Users

Dual users were defined as participants who self-identified as current users of combustible cigarettes and e-cigarettes. All participants reported currently using e-cigarettes.

#### 2.3.3. Vaping, Quantity of E-Juice Used, and Nicotine Use

Number of vaping days per month was reported as a categorical variable with response options: none, 1 to 2 days, 3 to 5, 6 to 9, 10 to 19, 20 to 29, and every day. Because most participants vaped every day, vaping days per month was dichotomized into two groups: every day users and less than every day users. Participants reported the amount of e-juice used per day (ml/day), which was multiplied by the number of vaping days per month. Nicotine use was defined using two measures: 1) nicotine level in e-juice and 2) total amount of nicotine per month (mg/month). The highest e-juice nicotine level used in a vape pen or advanced personal vaporizer was reported as: none, 1-3mg/ml, 4-11mg/ml, 12-17mg/ml, 18-24mg/ml, or more than 24mg/ml. The total amount of nicotine consumed (mg/month) was calculated by multiplying the e-juice nicotine level (mg/ml) by the amount of e-juice used per day (ml/day) and the number of days used per month.

#### 2.3.4. Initial Nicotine Exposure

Interest centered in how soon after waking a participant smoked or vaped. The question, “How soon after you wake do you smoke?” with standard response options from the Fagerstrom Test for Nicotine Dependence (FTND) was asked for two different time periods: prior to and after vaping initiation. The questionnaire also contained, “How soon after you wake do you vape?” with the standard FTND response options. For dual users, the time of earliest exposure after vaping initiation was determined by either the time of earliest exposure to nicotine from smoking or the time of earliest exposure to nicotine from vaping. The earliest time recorded was determined to be the time of initial nicotine exposure. For e-cigarette only users, the time of earliest exposure after vaping initiation was the time of first e-cigarette use after waking. For both dual and e-cigarette only users, the time of first exposure before vaping initiation was the first time of combustible cigarette use after waking. The average of each response option interval was calculated in minutes and the difference between the time of first exposure to nicotine in the morning after and prior to vaping initiation was assessed.

### 2.4. Statistical Analysis

P-values were calculated using nonparametric tests due to the non-normal distribution of the data and small sample size. Fisher’s Exact Test was used to analyze categorical variables, and the Kruskal-Wallis Test and Wilcoxon Signed Rank Test were used to analyze continuous variables. An exact multivariable logistic regression analysis was performed to assess the relationship between the dependent variable, classification of product users (e-cigarette only vs. dual user), and independent variables describing e-cigarette use behaviors [20].

Education level and nicotine level (mg/ml) were omitted from model consideration due to low frequency. All other statistically significant variables from Table 1 were considered for inclusion as independent variables in the regression model using a combination of forward and backward selection. The correlation between independent variables was assessed. When a correlation was found between variables, only the variable with the best-fit and lower p-value was kept in the model. All variables that had a p-value >0.05 were not included in the multivariable model [21].

The final regression model included the amount of e-juice used per month and time of first exposure to nicotine after waking as independent variables. Although both amount of nicotine used per month and amount of e-juice consumed per month were associated with being an e-cigarette only user compared to a dual user, there was a strong correlation between the two variables (Pearson’s r=0.598, p<0.0001). Due to the correlation, only amount of e-juice consumed per month was selected for inclusion with a better overall fit. Odds ratios and 95% confidence intervals are reported for the regression model. Due to the relative small units of measure (milliliters and minutes), the odds ratios for amount of e-juice consumed per month and minutes until first exposure after waking were converted from milliliters per month to milliliters per day and 1-minute to 5-minute intervals, respectively. All tests performed were two-tailed with a significance level of 5%. All statistical analyses and descriptive statistics were generated using SAS 9.4 [22].

## 3. Results

Participant characteristics as well as e-cigarette and tobacco use patterns are reported in Table 1. There was no significant difference in age, gender, race, or income between e-cigarette only and dual users. More e-cigarette only users had a college degree compared to dual users (p=0.03). Compared to dual users, e-cigarette only users consumed a significantly greater amount of e-juice per month (p=0.0004) and vaped every day in the past month (p=0.003). In addition, users of e-cigarettes only consumed higher levels of nicotine in e-juice (p=0.0009) and more nicotine (mg) per month (p=0.03) in e-cigarettes compared to dual users.

There was a significant difference in time of first nicotine exposure between e-cigarette only and dual users, with e-cigarette only users vaping an average of 26.6 minutes (SD= 22.0) after waking and dual users consuming nicotine an average of 9.6 minutes (SD= 8.0) after waking (p=0.0056). E-cigarette only users significantly delayed nicotine consumption after waking an average of 11.1 minutes (SD= 27.2) compared to time of first nicotine exposure prior to e-cigarette use initiation (p=0.0009). Although not significant, after e-cigarette use initiation dual users consumed nicotine an average of 6.8 minutes (SD= 24.4) sooner after waking compared to time of first nicotine exposure prior to e-cigarette use initiation (p=0.8). Between e-cigarette only and dual use groups, there was a significant difference in the change in time of first nicotine exposure before and after e-cigarette use initiation (p=0.04).

Results from the exact multivariate logistic regression model suggest the amount of e-juice consumed (ml/month) and the time of first nicotine exposure (minutes) after waking is significantly associated with being an e-cigarette only user. For every milliliter of e-juice used in a day, the odds were 1.50 times greater (95% CI= (1.11, 2.33)) that the user would only consume e-cigarettes (i.e., not be a dual user) when controlling for time of first nicotine exposure. Delay in first e-cigarette use after waking was also significantly associated with being an e-cigarette only user (OR=1.47 for every five-minute delay; 95% CI= (1.01, 2.86)) when controlling for amount of e-juice used. Although nicotine level of e-juice and total amount of nicotine consumed in a month significantly differed between dual users and e-cigarette only users, neither of these predictors contributed significantly to the model showing that the volume of e-juice consumed has a stronger effect than total nicotine consumed.

## 4. Discussion and Conclusions

Our findings indicate that even though e-cigarette only users purchase e-juice with lower nicotine levels, they vape more often as well as consume more e-juice and nicotine from e-cigarettes than dual users. However, e-cigarette only users’ initial use of nicotine after waking is significantly later compared to dual users, as well as compared to their own initial use of nicotine prior to e-cigarette initiation, suggesting e-cigarette only users may be less addicted to nicotine and/or have the potential of some harm mitigation by later use.

Some studies have found that users of combustible tobacco who switch to e-cigarettes report health benefits [23]. However, the health effects of e-cigarettes have not been well studied, and the long-term health risks potentially associated with vaping are still unknown. The time required for nicotine to be absorbed into the blood stream during e-cigarette use by experienced vapers is equivalent to combustible cigarette use, but other similarities and differences in nicotine delivery between combustible cigarettes and e-cigarettes need to be further explored (e.g., effects to mouth, throat teeth; general effects of inhaling different products) [24, 25]. In addition, future inquiry to better understand the impact of specific product use (e.g., individual tobacco products and the combined effects of dual use) on nicotine addiction is warranted.

Because marketing is influential in shaping public views and additional research needs to be conducted to more fully understand of the effects of e-cigarette use, the FDA should consider regulating marketing claims. For example, claims of cessation success or health benefits as well as messages targeting youth, even indirectly, need to be carefully assessed. Accurate marketing messages allow current and potential consumers to make more informed choices, even if potential long-term risks are unclear. If, as research evidence mounts, e-cigarettes are shown to have clear cessation benefits or eliminate dependence on nicotine, then FDA oversight, as with all cessation aides, would ensure safety standards are met for particular products and protect public health.

Future research should examine nicotine consumption levels of dual users and e-cigarette only users longitudinally to determine patterns across time. Some studies have begun to assess changes over time, but longer timeframes are needed to examine overarching change [26]. Additional assessments of delays in nicotine consumption by e-cigarette only users compared to dual users would also be useful. As this work moves forward, more attention needs to be devoted to agreed upon measures of e-cigarette use and nicotine consumption. Also, additional inquiry into the goals of dual users and e-cigarette only users is warranted. For example, to what extent are goals of smoking cessation, nicotine reduction, or other motivations being achieved? Further, as more evidence shapes knowledge of the health outcomes associated with e-cigarette use, assessments of harm reduction may be clearer.

Study limitations include a relatively small sample size and a focus in one metropolitan area as well as self-report measures and a potential recall bias in asking participants to remember how much they smoked in the past. Despite these limitations, our findings shed light on consumption patterns of dual and e-cigarette only users, adding to emerging understandings of vape shop patrons and their behaviors. This study found that e-cigarette only users vape more often and consume more e-juice overall than dual users. The long-term health effects of e-cigarette use are unknown and examining patterns of use and overall consumption is important as understandings of combustible cigarette cessation likelihood, level of nicotine consumed, and other differences by groups are developed.

## Figures and Tables

**Table-1.** Participant Characteristics

	E-Cigarette only n=67	Dual User n=ll	Total N=78	P-value
**Sex**				0.40
*Females*	22.7% (15)	36.4% (4)	24.7% (19)	
*Males*	77.3% (51)	63.6% (7)	75.3% (58)	
**Age**				0.60
*Mean (SD)*	30.3 (10.6)	28.9 (10.7)	31 (10.6)	
*Median (range)*	30.3 (19.7)	25.1 (7.2)	27.8 (17.1)	
**Race**				0.20
*White/Caucasian*	89.4% (59)	80% (8)	88.2% (67)	0.30^a^
*Black/African American*	3% (2)	0% (0)	2.6% (2)	
*Hispanic/Latino*	0% (0)	10% (1)	1.3% (1)	
*American Indian/Alaskan*	3% (2)	10% (1)	4% (3)	
*Asian*	1.5% (1)	0% (0)	1.3% (1)	
*More than one race*	3% (2)	0% (0)	2.6% (2)	
**Education**				0.03
*High School Graduate, GED, Vocational or Some College*	67.7% (44)	100% (11)	72.4% (55)	
*2-Year College Degree, 4-Year College Degree or Advanced/Professional*	32.3% (21)	0% (0)	27.6% (21)	
**Household Income**				0.20
*Less than $50,000*	33.9% (20)	60% (6)	37.7 (26)	
*More than $50,000*	66.1% (39)	40% (4)	62.3 (43)	
*Do not wish to disclose = 9*				
**Vaped every day in the past month**				0.003
*No*	3% (2)	36.4% (4)	7.7% (6)	
*Yes*	97% (65)	63.6% (7)	92.3% (72)	
**Nicotine Level**				0.0009
*No Nicotine (0 mg/ml)*	9% (6)	0% (0)	7.7% (6)	0.0003^b^
*Low (1-3 mg/ml)*	58.2% (39)	9.1% (1)	51.3% (40)	0.60^c^
* Medium (4-11 mg/ml)*	20.9% (14)	81.8% (9)	29.5% (23)	
* High (12-24 mg/ml)*	11.9% (8)	9.1% (1)	11.5% (9)	
**Amount of e-juice (ml/month)**				0.0004
*Mean (SD)*	208.0 (209.3)	45.3 (66.8)	185.0 (203.5)	
*Median (range)*	120 (0-900)	30 (0-232)	120 (0-900)	
**Amount of nicotine from vaping (mg/month)**				0.03
*Mean (SD)*	763.9 (922.2)	339 (501.2)	704.0 (885.3)	
*Median (range)*	480 (0-4,500)	225 (0-1,740)	450 (0-4,500)	
**Delay in initial nicotine use after waking** ^d^				0.04
*Mean (SD)*	11.1 (27.2)	−6.8 (24.4)	8.6 (27.4)	
*Median (range)*	0 (−72.5-74.5)	0 (−74.5-15)	0 (−74.5-74.5)	

a P-value comparing White/Caucasian to all other races.

b P-value comparing No Nicotine/Low and Medium/High nicotine levels.

c P-value comparing Nicotine (Low, Medium, and High) and No Nicotine.

d Measure defined as the difference in time (minutes) of the first exposure to nicotine after waking via smoking or vaping post e-cigarette use initiation and time of first exposure to nicotine after waking via smoking prior to e-cigarette use initiation.
